# Agriculture, Food Systems, and Nutrition: Meeting the Challenge

**DOI:** 10.1002/gch2.201600002

**Published:** 2017-03-03

**Authors:** Stuart Gillespie, Mara van den Bold

**Affiliations:** ^1^ International Food Policy Research Institute 2033 K Street N.W. Washington DC 20006 USA

**Keywords:** agriculture, food systems, nutrition, South Asia, sub‐Saharan Africa

## Abstract

Malnutrition is a global challenge with huge social and economic costs; nearly every country faces a public health challenge, whether from undernutrition, overweight/obesity, and/or micronutrient deficiencies. Malnutrition is a multisectoral, multi‐level problem that results from the complex interplay between household and individual decision‐making, agri‐food, health, and environmental systems that determine access to services and resources, and related policy processes. This paper reviews the theory and recent qualitative evidence (particularly from 2010 to 2016) in the public health and nutrition literature, on the role that agriculture plays in improving nutrition, how food systems are changing rapidly due to globalization, trade liberalization, and urbanization, and the implications this has for nutrition globally. The paper ends by summarizing recommendations that emerge from this research related to (i) knowledge, evidence, and communications, (ii) politics, governance, and policy, and (iii) capacity, leadership, and financing.

## 1 Introduction

Malnutrition is a global challenge with huge social and economic costs, and the biggest risk factor for the global burden of disease.^1^ One in three people are affected, and virtually every country on this planet is facing a serious public health challenge due to malnutrition.^2^, ^3^ Many countries are dealing with a “triple burden” of energy and micronutrient deficiencies, co‐existing with rising rates of overweight and obesity.^4^, ^5^

In terms of undernutrition, 159 million children under five years of age are stunted (low height‐for‐age), while 795 million people are hungry.^1^ Micronutrient deficiencies afflict two billion people worldwide. Iron deficiency alone affects more than one and a half billion people worldwide; in Africa and Southeast Asia, two‐thirds of preschoolers and around half of all pregnant women are anemic.^6^ Vitamin A deficiency affects 250 million preschool‐age children, blinding up to 500 000 of them, half of whom will die shortly after losing their vision.^7^ A similar number of children have insufficient iodine intake, which significantly impairs their cognitive development.^8^

The consequences of malnutrition are massive, pervasive, and often hidden. Malnutrition (in some form) is a cause of 45% of all deaths of children under five years of age, amounting to over three million deaths each year. It stunts growth, erodes child development, reduces the amount of schooling children attain, and increases the likelihood of poverty in adulthood. It persists through the life cycle and across generations, with underweight mothers more likely to give birth to underweight children. Undernutrition reduces global gross domestic product (GDP) by up to USD 2 trillion per year—the size of the total economy of Africa south of the Sahara.^9^ Annual GDP losses due to malnutrition average 11% in Asia and Africa—greater than the loss experienced during the 2008–2010 financial crisis.^1^

In addition to undernutrition, the health landscape in all regions of the world is being drastically altered by an epidemic of another form of malnutrition: overweight/obesity. As of 2010, undernutrition affected 2.1 billion people worldwide and caused 3.4 million deaths globally.^10^ Currently, 42 million children are overweight and obese, following a dramatic 47% rise in prevalence between 1980 and 2013.^10^, ^11^ Obesity is increasing across the board—in most countries, in both urban and rural settings, and across socio‐economic levels, including the poorest—raising the risk of non‐communicable diseases (NCDs), including type 2 diabetes, hypertension, dyslipidemia, and various cancers.^12^, ^13^, ^14^ Child obesity is of particular concern, as it exacerbates risk factors for NCDs in adulthood, especially in those who have poor linear growth.^4^, ^15^

Different forms of malnutrition co‐exist in the same communities and households, in part reflecting similar basic causes (poverty, lack of education, and poor physical and economic access to healthy diets). A study conducted in urban Kenya found that a large proportion of mothers who were overweight (43%) or obese (37%) had stunted children.^16^ Multiple forms of malnutrition co‐existing within the same household have been observed in other countries, and they may also co‐exist in the same individual, for example, when a stunted child becomes overweight or obese.^17^, ^18^, ^19^

## 2 What Causes Malnutrition?

**Figure 1:**
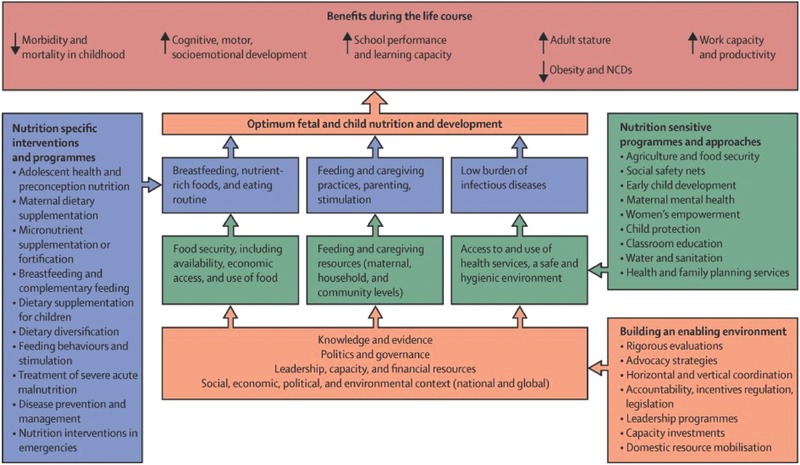
Framework for actions to achieve optimum fetal and child nutrition and development.^21^

The conceptual framework pioneered by UNICEF in the early 1990s and further adapted in the 2013 Lancet Nutrition Series (**Figure** **1**) highlights the drivers of nutritional status at different levels, and the types of sectoral responses that are required to prevent, or respond effectively to, malnutrition. The framework was originally focused on undernutrition; in this iteration, it is oriented toward optimum fetal and child nutrition and development.

Malnutrition can be viewed as an outcome of dysfunctional interactions between different systems: the agri‐food system, the environmental system, the health system, and, crucially, the system of individual and household decision‐making.^22^

Another complementary conceptual approach is to consider “environments.” At the base of Figure 1, underpinning the set of drivers of nutritional status that operate at different levels, lies an “enabling environment for nutrition.” This can be defined as the “wider political and policy processes which build and sustain momentum for the effective implementation of actions that prevent or reduce (mal)nutrition.”^23^ Key ingredients of such environments include (a) knowledge, data, and evidence and its effective framing and communication, (b) political commitment, effective governance, and sound policy, and (c) leadership, capacity, and financing.^24^ Environments may be enabling (regarding their effects on nutrition), they may be neutral, or they may be “disabling.” Like undernutrition, obesity is a complex, multifactorial problem with genetic, lifestyle, cultural, medical, and social causes that have been fueled by rapid economic, societal, and cultural changes.^25^ Swinburn et al. first coined the term “obesogenic environment” to refer to “an environment that promotes gaining weight and one that is not conducive to weight loss” within the home, workplace, or society. We will return to the enabling environment framework in the final section.^26^

As malnutrition is the final outcome of a combination of determinants, clustered into food, health, and care, it requires responses from a range of sectors: food security, public health, water, sanitation and hygiene, and social protection. Nutrition is not itself a sector, but it is dependent on actions that originate from these sectors if it is to be effectively and sustainably addressed. The framework in Figure 1 highlights this multisectorality, but also shows the multi‐level nature of response, differentiating direct (nutrition‐specific) interventions, usually delivered by the health sector, and indirect (nutrition‐sensitive) programs implemented by a variety of sectors, both of which are underpinned by enabling policy environments.^21^, ^27^ Even if the recommended package of nutrition‐specific interventions put forward by the Lancet Nutrition Series was scaled up to 90% population coverage in the 34 countries with the highest burden of undernutrition, child stunting would fall by only 20%.^28^ This means that efforts to scale up nutrition‐specific interventions need to be paired with investments in nutrition‐sensitive development programs and policies that address the underlying drivers of malnutrition.

Agriculture is obviously a key sector, but again it is important to understand interactions with other sectoral actions (and to seek to turn negatives into positives). Agriculture needs to work in harmony with other sectors to maximize its impacts on nutrition. For example, social protection can protect the nutrition and health of poor smallholder households as they grapple with seasonality and climate shocks and stresses. Improved water, sanitation, and hygiene (WASH) can increase the nutritional benefits of agricultural programs and policies aimed at improving diets by reducing disease and enhancing nutrient absorption. And linkages between local agricultural production and school feeding may generate win–win benefits: income for small producers and their families, and nutrition and cognitive gains (and likely future income) for school‐age children.

While recognizing this essential multisectorality of nutrition, we will focus our attention in this paper on agriculture and food systems and their relationship with nutrition. The next section focuses on the evidence base with regards to linkages between agriculture and undernutrition, as this has been where much past work has been concentrated. We focus particularly on the findings of recent studies and on reviews in the public health and nutrition literature that contextualize this work with regard to older studies that emerged in the 1990s–2000s. This is followed by a section that broadens the review to food systems and malnutrition, with a focus on diets and obesity. Drawing on the material we review, we conclude with a set of recommended actions to enhance the nutrition‐sensitivity of agriculture and food systems.

## 3 Agriculture and Nutrition

Agriculture produces the food people eat and is the primary source of livelihood (employment, income) for most the world's poor, who, in turn, are most vulnerable to ill health and malnutrition. Agricultural development has enormous potential to make significant contributions to reducing malnutrition and associated ill health. With its close links to both the immediate causes of undernutrition (diets, feeding practices, and health) and its underlying determinants (such as income, food security, education, access to WASH and health services, and gender equity), the agriculture sector can play a much stronger role than in the past in improving nutrition outcomes.^29^, ^30^

**Figure 2:**
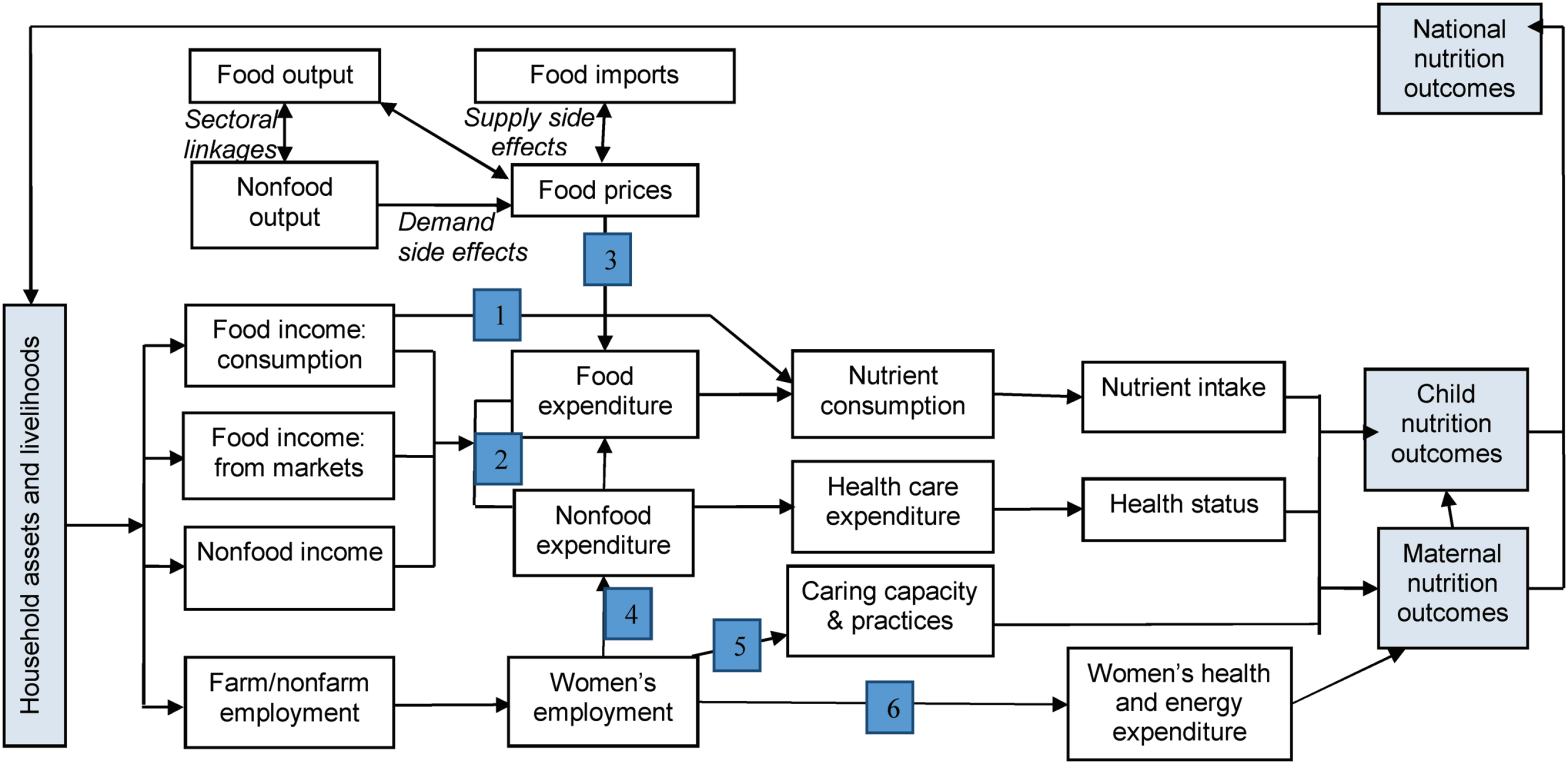
The TANDI framework conceptualizing pathways and links between agricultural livelihoods and nutrition outcomes.^31^, ^32^

In recent years, one particular framework, developed for the Tackling the Agriculture–Nutrition Disconnect in India (TANDI) project and shown in **Figure** **2**, has been used to conceptualize pathways through which the agriculture sector may impact nutrition outcomes.^31^, ^32^ This is complementary to the more inclusive “global” framework (Figure 1), as it only seeks to unpack drivers and links between one sector—agriculture—and nutrition.

Six pathways linking agriculture and nutrition are highlighted in this framework, numbered in Figure 2 and summarized here:

- Pathway 1: Agriculture as a source of food for household consumption: the most direct pathway by which household agricultural production translates into consumption (via crops cultivated by the household).
- Pathway 2: Agriculture as a source of income for food and nonfood expenditures: agriculture generates income (via wages earned or through sale of food produced), which is translated into expenditure on nutrition‐enhancing goods and services (including health, education, and social services).
- Pathway 3: Effects of agriculture policy and food prices on food consumption: this link involves a range of supply‐and‐demand factors that affect food prices, which in turn affect purchasing power of net buyers.
- Pathway 4: Effects of women's employment in agriculture on intrahousehold decision making and resource allocation: agricultural labor conditions can influence the empowerment of women and thus their control over nutrition‐relevant resources and decision making, particularly regarding food and healthcare.
- Pathway 5: Effects of women's employment in agriculture on childcare and child feeding: relates to the challenges that heavy and prolonged female workloads in agriculture present to ensuring adequate care for young children.
- Pathway 6: Effects of women's employment in agriculture on their own nutritional and health status: relates to the energy‐intensive nature of agricultural labor and effects on maternal nutritional and health status, and to related health hazards (including exposure to pathogens through waste water irrigation and/or livestock and poultry in the homestead).

Though these pathways are depicted separately, they overlap and interact. Pathways 1 and 2, for example, relate to the “separability” hypothesis.^29^, ^33^ Like other productive sectors, agriculture generates income that can be spent on nutrition‐enhancing goods and services (Pathway 2), although agriculture is generally a more important source of income for the poor and undernourished, both directly, and through the so called “multiplier effects” on other sectors.^34^ Because of various market failures, however, farmers may choose to grow food that they consume (Pathway 1), thus rendering agriculture a special sector for nutrition, but also opening up complex dynamic policy tradeoffs.^29^, ^35^ Pathway 3 highlights the macroeconomic linkages between agricultural production conditions and food prices, which can drive consumption decisions. Pathways 4–6 go beyond price and income to focus on the linkages between child undernutrition and maternal socioeconomic and nutritional status. Agricultural production conditions can affect women's decision‐making power and control of nutrition‐relevant resources (Pathway 4), as well as their ability to manage the care of young children which is of huge importance for nutrition (Pathway 5).^36^ At this point we can again see important trade‐offs between several pathways. The TANDI initiative, for example, has shown that if a rise in the demand for female agricultural labor is not matched by enhanced decision‐making power and control of household resources (including time), both women and children's nutritional status may suffer. Finally, Pathway 6 addresses the possibility that the often arduous and hazardous conditions of agricultural labor in India pose substantial risks for maternal nutritional status and an intergenerational transmission of undernutrition.

In Box 1, we highlight a recent review of agriculture and nutrition links in six countries (three in South Asia, three in sub‐Saharan Africa), as part of the Leveraging Agriculture for Nutrition in South Asia (LANSA) and Leveraging Agriculture for Nutrition in East Africa (LANEA) initiatives. These reviews all applied the TANDI framework in a set of structured evidence reviews.^37^

Box 1: **The LANSA and LANEA Initiatives**

The LANSA and LANEA used the TANDI framework to map evidence against the agriculture–nutrition pathways in six countries in South Asia and East Africa. Further details on methodology of these reviews can be found in the cited papers here, and in the following reviews: Gillespie et al. (2015); Hodge et al. (2015); van den Bold et al. (2015).^38^, ^39^, ^40^

**South Asia**. The work in India found that, despite a weak evidence base (i.e., a lack of studies with strong causal identification, limited inclusion of anthropometry, or micronutrient status as outcomes, and limited inclusion of dietary diversity indicators at individual level), the agricultural sector contributes to dietary patterns of farm households, relative food prices as well as particular food items, and to income and expenditure, but is much weaker in relation to the role of women in agriculture, particularly in relation to the impact on women's time use.^29^ In Pakistan, Balagamwala and Gazdar (2013) found that more analysis is required in Pakistan around mediating factors within these pathways, such as gender relations, preferences and behaviors of individuals and households, political priorities, and organizational effectiveness (including for example, quantity and quality of public investment), and access to land.^41^ In Bangladesh, Yosef et al. (2015) found that while more research is needed across all pathways, it is particularly lacking with regard to agriculture as a source of income (pathway 2), and pathways 4–6 with regard to women's participation in agriculture and how this impacts nutrition and health in their households.^42^ They also called for more explicit measurement of related outcomes in terms of women's empowerment and dietary diversity.

**Sub‐Saharan Africa**. Similarly, LANEA mapped evidence across agriculture–nutrition pathways in East Africa, in Kenya, Ethiopia, and Uganda, countries where agriculture also continues to play an important role. In all three countries, most studies tended to focus on pathway 1 (agriculture as a source of food), with particularly limited research and evidence on pathway 6 (women's participation in agriculture and their own nutrition and health status). In Ethiopia, studies showed that it was difficult for households to achieve food security solely through household production. Land ownership had a positive impact on food security, with women's land tenure security particularly important in rural areas. Female‐headed households were more likely to experience a decrease in asset holdings due to volatility in food prices, putting pressure on women's time. Authors also found that net purchasers of food were more vulnerable to food price increases, particularly the urban poor, and that, lastly, adolescent boys were favored over adolescent girls in allocation of household resources.^43^ In Kenya, interventions to improve vegetable, animal source foods (ASF), and fruit production produced mixed results, but ownership of livestock and milk consumption was associated with better nutrition outcomes. Poorer households however faced challenges with intensive dairy production due to high input costs. Although income from on‐ and off‐farm employment and food‐for‐work was associated with better food security and variety and quantity of foods consumed, it did not always lead to improvements in nutritional status, particularly if health and child care practices were sub‐optimal. With regards to food prices, especially households without access to food produced locally struggled to achieve adequate food consumption, and it was difficult for households to meet nutritional requirements with their own food production, particularly when production was influenced by seasonality. Women's employment in agriculture was found to have positive impacts on nutrition in the household when women had decision‐making power over resource allocation.^44^ In Uganda, evidence from randomized controlled trials showed positive impacts from biofortified crops, including orange‐fleshed sweet potato, on vitamin A status among women and children. Ownership of livestock was associated with better household food security in Kampala. Evidence also showed mixed impacts on the links between women's empowerment, intrahousehold decision‐making, and better nutrition outcomes.^45^

In spite of recent positive trends in commitments and investments in increasing the nutrition‐sensitivity of agriculture, examples of success in improving maternal and child nutrition documented through standardized rigorous methods has only started to emerge.^46^, ^47^, ^48^, ^49^ To date, there is still limited evidence that agricultural interventions are benefiting nutrition or that agricultural growth consistently leads to nutritional improvements.^50^, ^51^ In many low‐ and middle‐income countries, large changes in agricultural policy and practice have generated relatively small changes in nutrition.^32^, ^52^

While agriculture has relatively high economic returns to investment, and has an immense potential to reduce undernutrition, it is well‐known that an improvement in food production or consumption does not necessarily lead to improvements in health and nutrition outcomes.^50^, ^53^ As Figure 1 shows, poor quality of health services, disease (possibly agriculture‐related), lack of adequate sanitation and hygiene, and lack of women's empowerment can subvert agriculture's positive impacts on nutrition outcomes. As a consequence, the potential of agriculture to reduce undernutrition is not being realized in many countries.^29^, ^40^, ^41^, ^50^, ^54^

Focusing specifically on agricultural interventions—primarily related to home gardening and animal/dairy production—evidence is mixed in terms of their impacts on nutrition‐related indicators. While several studies in the late 2000s—mostly in South Asia and Africa south of the Sahara—documented positive impacts on intermediary nutrition outcomes, such as dietary diversity, household production and consumption, and child and maternal intake of “target” foods and micronutrients, evidence of impact on nutrition outcomes—particularly child anthropometry and micronutrient status—was much more limited, except in relation to vitamin A intake and status.^50^, ^55^, ^56^, ^57^, ^58^, ^59^ A more recent review of eight studies by Carletto et al. (2015) examined the relationship between agricultural production (crops and livestock), dietary diversity in households, and child and maternal diet and nutrition outcomes in sub‐Saharan Africa and South Asia, and found that, together, these studies demonstrated that household agricultural production can directly influence household dietary patterns and the nutritional status of household members, but the extent of impact depends on a variety of factors including location, commodities, and the role of livestock.^60^

Several studies have pointed to improved impacts on nutrition if agricultural interventions are targeted to women and when specific work is done around women's empowerment (for example, through behavior change communication), mediated through women's time use, women's own health and nutrition status, and women's access to and control over resources as well as intrahousehold decision‐making power.^50^, ^61^, ^62^, ^63^, ^64^, ^65^, ^66^, ^67^

Box 2: **The rice‐wheat Green Revolution in Asia: a mixed legacy?**

Following the Second World War, increasing food production was considered fundamental to fighting hunger, reducing social inequities, and lifting families out of poverty.^68^ In the 1960–1970s, the Green Revolution investment in high‐yielding varieties of wheat and rice massively ramped up cereal yields in Asia and Latin America and helped save millions of lives.^69^ Agricultural growth has driven rapid economic growth in many countries with widespread benefits to millions.

And yet there is a dark side to this legacy in the hyper concentration of agricultural policy and incentives on a few staples and on calories, as opposed to nutrient diversity. Nutrient‐dense crops like pulses, fruits, and vegetables have been marginalized. Millions of smallholders who produce food but are net buyers are hit by price volatility and sharp hikes. Just three food crops—rice, maize, and wheat—now provide nearly two‐thirds of global dietary energy intake.^68^

As nutrition has continued to be viewed as a health concern, for the health sector, agriculture has increasingly become skewed to producing animal feed, biofuels, and industrial ingredients for processed food products (e.g., sugar‐sweetened beverages, ready‐to‐eat meals, and snacks).

Surprisingly little is known about the nutrition impacts of Asia's Green Revolution, and much of what has been written is speculative at best. In a pioneering study, Headey and Hoddinott (2016) sought to fill this gap by creating a multi‐round district level panel dataset that links changes in nutrition outcome data with agricultural sample survey data in Bangladesh for the period 1996–2011, a period in which rice yields rose by 70% (Bangladesh being a relatively late‐adopter of GR technologies). In sum, the authors find that rice yields predict an earlier introduction of complementary foods to young children (mostly rice) as well as increases in weight‐for‐height. But they find no evidence of any associated improvement in height‐for‐age (and thus stunting) or diet diversity. Further nutritional impacts will require a diversification of the Bangladeshi food basket through both supply and demand side interventions.^70^

In the most recent review, Fiorella et al. (2016) examined 42 evaluations of agricultural interventions and their impact on child and maternal nutrition, and found that further information regarding agricultural programs' impact on time burdens, income, and expenditures would be useful in evaluation findings. They called for greater consideration of political, economic, environmental, and cultural factors in the assessment of particular projects (such as land tenure, weather patterns, resource access, and government policies), as these can substantially impact their outcome.^71^

Lack of evidence of impact however is not equivalent to lack of impact. Weak program design and implementation, and/or a lack of methodological rigor in designing evaluations and studies have limited the evidence base.^50^, ^57^, ^58^, ^59^, ^72^, ^73^

As Herforth and Ballard (2016) state: “[t]he evidence base for impact of agriculture on nutrition is bounded by what is measured.”^74^ Reviews have highlighted the following limitations: (i) lack of rigorous evaluations (i.e., adequate sample size, appropriate comparison groups), (ii) few interventions targeted to the first 1000 days (window of opportunity for impacting child nutrition), (iii) weak design and implementation in terms of programs' nutrition‐sensitivity (such as use of food expenditure or household calorie consumption as indicators but no use of anthropometry or dietary diversity indicators).^29^, ^50^ As Herforth and Ballard (2016) suggest, there is a need to consider what is appropriate and feasible regarding indicators of agriculture's impact on nutrition. Is it reasonable to expect impacts on stunting, and to be able to attribute them to an agricultural intervention? They call for a wider array of indicators to capture change along impact pathways, such as women's empowerment, food and health environments, and dietary quality (we return to this in the final section).^65^

## 4 Food Systems, Dietary Change, and Obesity

Food systems are changing rapidly.^75^ Globalization, trade liberalization, and rapid urbanization have led to major shifts in the availability, affordability, and acceptability of different types of food, which has driven a nutrition transition in many countries in the developing world.^76^, ^77^, ^78^, ^79^

Globalization generates marketing systems that require food production to be intensified and standardized. Food production has become more capital‐intensive and supply chains have grown longer as basic ingredients undergo multiple transformations before the final product.^80^ Value chains shift power from producers to retailers and supermarkets. Standardization benefits larger suppliers rendering global markets more difficult to access for smallholder farmers. Family agriculture and associated (agro)biodiversity is being marginalized, though smallholders continue to play a crucial role in supplying local markets with fresh and affordable agricultural produce.

The consequences of an increasing globalization of value chains reach well beyond the agricultural production system: the emergence of fast food outlets and supermarkets, the intensification of advertising and marketing of comparably cheap industrialized products, and foreign direct investment in developing countries and accelerating urbanization, have translated into major and rapid shifts in dietary patterns. The consumption of low nutritional quality, energy‐dense, ultra‐processed food and drinks, and fried snacks and sweets has risen dramatically in the past decade.^79^ Aggressive marketing of such foods by transnational companies has coincided with a shift from home‐prepared/home‐based meals to pre‐prepared/ready‐to‐eat meals.^80^, ^81^ Combined with increasingly sedentary lifestyles, rates of overweight and obesity and associated diet‐related chronic diseases have skyrocketed.^54^, ^82^, ^83^, ^84^, ^85^, ^86^

The diet transition plays out against this backdrop, and it moves through different phases, as incomes tend to rise. As incomes rise, the urban poor and emerging middle‐class households tend to reduce their consumption of cereals, roots, and tubers while increasing demand for refined grains and flours, sugar, salt, and fats. Demand for processed, convenience/fast foods at supermarkets, restaurants, and informal street foods rises. For middle‐class population groups, demand for fruits, vegetables, and ASF, such as dairy, poultry, eggs, meat, and fish, strongly increases.^85^ In high‐ and middle‐income countries, consumption of healthier foods has grown in the past two decades, but particularly in low‐income countries, consumption of less healthy foods, such as processed meats and sugars, is rising even faster.^87^

Pingali et al (2016) suggest a three‐step typology of agri‐food systems that reflect stages of structural transformation that countries go through, and the need to articulate different strategies (to enhance agriculture's contribution to diet quality and nutrition) for each typology:^88^

1. Low‐productive agricultural systems (e.g., sub‐Saharan Africa) need yield enhancement, while maintaining production diversity and ensuring equitable conditions for working women.
2. Modernizing systems (e.g., Asia) need to diversify away from conventional staples to focus more on legumes and micronutrient‐rich foods.
3. Commercialized systems (e.g., Europe, North America) need to regulate ultra‐processed foods and seek to reduce consumers' sugar and salt consumption.

Similarly, the Global Nutrition Report has a more fine‐grained differentiation of five stages of food system “evolution”:^89^

1. Rural food systems (low agricultural productivity, high reliance on staples (e.g., Bangladesh, Ethiopia).
2. Emerging food systems (more urbanized, still reliant on staples (e.g., Pakistan, Thailand).
3. Transitioning food systems (e.g., Brazil, Malaysia).
4. Mixed food systems (moderate productivity, urbanization, low dependence on staples (e.g., Germany, Italy).
5. Industrial food systems (highly urbanized, low dependence on staples (e.g., USA, Sweden).

For each typology, a series of indicators can be used to measure four types of food system outcomes: food affordability, dietary diversity, health and nutritional status, and environmental sustainability. Different systems have different requirements if they are to be nutrition‐friendly and sustainable.^11^ Industrial systems need to increase fresh food consumption and rebalance protein sources away from certain animals; mixed systems need to reduce packaged food consumption; transitioning systems need to increase productivity and production diversity; emerging systems must reduce the “double burden” through more affordable, healthy food, in an environmentally sustainable way, and rural systems need to focus on improving productivity and ensuring food security.^11^

In addition to applying a nutrition lens to food systems, it is important to understand how they are increasingly threatened by (as well as contribute to) ongoing environmental trends, including global warming, desertification, and the increasing use of food crops for nonfood purposes. Increasing demands for energy‐intensive products also exacerbate environmental impacts of food value chains: industrial agriculture, intensifying production of high‐yield starchy staples through monoculture agriculture, leading to significant loss of food biodiversity; excessive use of agricultural chemicals to extract more dietary energy from every hectare while contaminating the very food it produces, along with groundwater and the soil; and the greenhouse gas emissions from livestock industries to feed the ever‐increasing demand for meat and dairy products.^90^ Weather‐related shocks linked to climate change may increase harvest failures, driving world food prices.

## 5 Meeting the Challenge

Over the last five years there has been a flurry of activity in terms of research and policy engagement on agriculture, food systems, and nutrition. In addition to the TANDI, LANSA, and LANEA initiatives described in this review, the CGIAR's Agriculture for Nutrition and Health (A4NH) program, Transform Nutrition, the World Bank, FAO, Action Contre la Faim, Save the Children and the UN Standing Committee on Nutrition have all been active in commissioning relevant studies, reviews, and recommendations.^91^, ^92^, ^93^, ^94^, ^95^, ^96^, ^97^, ^98^ Most recently, the three Global Nutrition Reports and the Global Panel on Agriculture and Food Systems for Nutrition flagship report “Food systems and diets: facing the challenges of the 21st century” have reviewed the evolving landscape. Recommendations from this work are similar and reinforcing.^1^, ^2^, ^11^, ^99^

**Figure 3:**
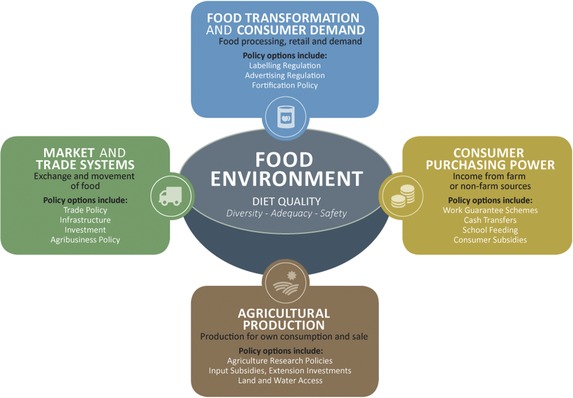
How agricultural and food system policies link to diet quality as a measure of good nutrition, including policy options.^100^

In this final section, we structure and summarize these main recommendations, using both the enabling environment three‐domain framework described earlier, along with the Global Panel's categorization of four clusters of policy options (see **Figure** **3**).^24^ Written in bullet form, these are largely recommendations for policymakers and investors in nutrition, toward creating and sustaining enabling policy and institutional environments for agri‐food systems to generate nutritional benefits.

1. Knowledge, evidence, and communication.

The first domain of the enabling environment framework comprises data, evidence, and knowledge (both of the nature of the “agriculture–nutrition disconnect” in this case and the potential solutions/responses), along with approaches to framing and communicating this knowledge to those who can use it. The priority now—as reflected in most of the recommendations below—is to generate and use knowledge of “what works at scale” and knowledge of how change can be catalyzed and sustained.

- Identify and embed appropriate nutrition‐relevant indicators and metrics, including (a) indicators of inputs, processes, and outcomes of agri‐food systems, and (b) collection of nationally representative integrated data across agriculture, food systems, nutrition, and health that reveal interactions and linkages.
- Use such data to progressively apply a nutrition lens to food systems and value chains, where “value” is no longer simply monetary. Such a nutrition lens would need to be bifocal—on the one hand reviewing likely implications and impacts on undernutrition (including micronutrient deficiencies) for vulnerable groups, and on the other, reviewing likely impacts on overweight and obesity.
- Evaluate programs, document “stories of change” and show what's possible (including “low hanging fruits,” and high‐impact, rapid‐return actions) to build demand. Highlight the “win–win” synergies (e.g., linking small‐scale local production with school feeding initiatives).
- Generate evidence on how to scale up and sustain nutrition‐sensitive actions.
- Raise awareness (using relevant media and communications channels) and generate demand for diets and food systems that are sustainable and healthy. Agriculture should be perceived to be not solely about food and feeding, but also about nutrition and nourishing.
- Support/fund rigorous monitoring of nutrition effects (pathways and outcomes) of agricultural investments, and more and better operational research and impact evaluations.
- Carry out more focused research to reveal trade‐offs and potential synergies of nutrition‐sensitive agriculture.
- Strengthen feedback and evidence‐to‐action loops so that lessons are learned and applied progressively.
- Link improved monitoring data with transparent systems of accountability and thus ultimately with action/change (via policies and clear roles and responsibilities of actors).
- 2. Politics, governance, and policy.

This second domain refers to the politics, institutional arrangements, policy and program decision‐making. The Global Panel on Agriculture and Food Systems for Nutrition have developed a simple framework (Figure 3) that shows the food environment (relating to diet quality, in terms of diversity, adequacy, and safety) as the necessary focus of agricultural and food system policies if they are to benefit nutrition. They further differentiate four types of policy options to this end: agricultural production, market and trade systems, food transformation and consumer demand, and consumer purchasing power. We use this structure here to summarize policy recommendations emerging from the literature reviewed, and the initiatives described, in this paper.

**Policies** need to:

- Have clearly defined objectives that derive from a comprehensive assessment and analysis of nutritional gaps and weaknesses in the food system.
- Provide/ensure institutional and policy environments, processes and incentives that foster appropriate forms of collaboration across nutrition‐relevant sectors (such as agriculture, health, education).
- Ensure clear and transparent systems of accountability at all levels e.g., developing scorecards.
- Embody mechanisms, principles, and processes that incentivize decisions, actions, and practices which are known to benefit nutrition, such as those shown in Figure 3 and described below.

**Agricultural Production**

- Align agricultural research investments to support nutritional improvement, such as more research on fruits and vegetables, animal source foods, nuts, and seeds.
- Find a balance between supporting agricultural producers to connect with globalized value chains and supplying traditional local markets with diverse, fresh foods.
- Promote and support more diverse production systems to include locally developed and adapted crop and animal varieties as well as input methods.
- Promote and support environmentally sustainable production, diversification, and improved productivity and availability of nutrient‐dense foods and small‐scale livestock.
- Improve and protect women's agency and control over resources, including time.

**Market and Trade Systems**

- Expand market (physical) access for vulnerable groups, particularly for nutritious foods, and for social protection/safety nets.
- Improve (infrastructure for) processing, storage, and preservation to retain nutritional value and food safety, to reduce seasonality and postharvest losses, and to make healthy foods convenient to prepare.

**Consumer Purchasing Power**

- Manage food price volatility (protect economic access for vulnerable groups).
- Improve nutritional quality of institutional diets e.g., in schools and hospitals.

**Food Transformation and Consumer Demand**

- Improve demand and consumption of fruits and vegetables, legumes/pulses, nuts and seeds, high‐protein, micronutrient‐dense grains, and safe milk.
- Promote development and use of national food‐based dietary guidelines to guide policy.
- Replace saturated and trans‐fats with unsaturated fats, and reduce high‐calorie, nutrient‐poor sugary drinks and salty snacks.
- Restrict advertising, marketing, and commercial promotion of unhealthy, low‐nutrient, and ultra‐processed foods.
- Control labeling of foods to ensure claims are evidence‐based.
- Prioritize the improvement of diet quality of young children, adolescent girls, and women, including animal source foods (fish, meat, eggs, and dairy).

Another useful summary set of policy actions—targeting obesity in particular—and which overlap with those listed above, is provided in **Table** **1** with the NOURISHING framework.^101^

**Table 1:** The NOURISHING framework^102^

**Domain**		**Policy area**	**Examples of potential policy actions**
**Food environment**	**N**	Nutrition label standards and regulations on the use of claims and implied claims on foods	e.g., nutrient lists on food packages; clearly visible “interpretive” and calorie labels; menu, shelf labels; rules on nutrient and health claims
	**O**	Offer healthy foods and set standards in public institutions and other specific settings	e.g., fruit and vegetable programs; standards in education, work, health facilities; award schemes; choice architecture
	**U**	Use economic tools to address food affordability and purchase incentives	e.g., targeted subsidies; price promotions at point of scale; unit pricing; health‐related food taxes
	**R**	Restrict food advertising and other forms of commercial promotion	e.g., restrict advertising to children that promotes unhealthy diets in all forms of media; sales promotions; packaging; sponsorship
	**I**	Improve the nutritional quality of the whole food supply	e.g., reformulation to reduce salt and fats; elimination of trans fats; reduce energy density of processed foods; portion size limits
	**S**	Set incentives and rules to create a healthy retail and food service environment	e.g., incentives for shops to locate in underserved areas; planning restrictions on food outlets; in‐store promotions
**Food system**	**H**	Harness the food supply chain and actions across sectors to ensure coherence with health	e.g., supply‐chain incentives for production; public procurement through “short” chains; health‐in‐all policies; governance structures for multi‐sectoral engagement
**Behavior‐change communication**	**I**	Inform people about food and nutrition through public awareness	e.g., education about food‐based dietary guidelines, mass media, social marketing; community and public information campaigns
	**N**	Nutrition advice and counselling in health‐care settings	e.g., nutrition advice for at‐risk individuals; telephone advice and support; clinical guidelines for health professionals on effective interventions for nutrition
	**G**	Give nutrition education and skills	e.g., nutrition, cooking/food production skills on education curricula; workplace health schemes; health literacy programs

**Programs** need to:

- Assess context.
- Consider the full range of pathways between agriculture (as a livelihood) and nutrition‐relevant outcomes (not only child stunting), especially pathways in which women are significantly engaged.
- Incorporate explicit nutrition objectives and indicators into the design of agriculture programs, and track and mitigate potential harms.
- Target the vulnerable and improve equity through participation, access to resources, and decent employment. Locate any action in a broader political perspective e.g., in relation to women's access to land, employment, health service, and education.
- Relatedly, apply a gender lens to assess how impacts of agriculture on nutrition may be mediated by women's roles in agriculture. Seek opportunities to strengthen women's power, agency and control of resources.
- Incorporate nutrition education and behavior change communication.
- Seek to do “double duty.” As stunting (undernutrition) predisposes to overweight in later life, both forms of malnutrition need to be viewed together. Programs to address undernutrition must not put too much emphasis on quantity of calories and weight gain, while anti‐obesity campaigns must avoid unintended consequences for undernutrition.
- 3. Capacity, leadership, financing.

The third domain of the “enabling environment” framework highlights the importance of capacity (at individual, organizational, and systemic levels) and financial resources to strengthen the nutrition‐sensitivity of agriculture.^24^, ^98^ Recommended actions include:

- Develop capacity for integration of disciplines (agriculture, health, nutrition) at different administrative levels (e.g., through cross‐disciplinary training, provision of tools to help communicate and work across sectors, disciplinary integration of course curricula).
- Develop mid‐level (district‐level) operational and strategy capacities. Training and education needs to be strengthened with regard to agriculture's linkages to nutrition.
- Cultivate leadership (transformational capacity). Nutrition champions, policy entrepreneurs, and civil society activists at all levels need to be supported and encouraged. Cross‐sectoral “lateral” leadership is needed to bridge sectoral divides (e.g., between agriculture and health).
- Clarify financing—budgetary reallocations and/or increased funding—for nutrition‐sensitive agri‐food systems (as part of a wider national costing exercise on nutrition).

## 6 Conclusions

Malnutrition kills millions and erodes the potential of billions. Poor diets and malnutrition are by far the biggest contributor to the global disease burden. As the most important source of livelihood for most nutritionally vulnerable people on the planet, agriculture is not doing enough to turn this situation around. In this paper, we have summarized the evidence for this agriculture–nutrition disconnect and highlighted policy and programmatic options for addressing this global challenge, drawing especially on research and discourse from the last five years or so in the public health and nutrition literature. The focus is on malnutrition, not simply undernutrition, as obesity is now epidemic in many countries. Because of this, the scope needs to be broadened to food systems at large, going well beyond agriculture.

Leveraging agri‐food systems for nutrition implies (a) creating and strengthening institutional and policy environments (including accountability systems) that enable agriculture and food systems to support nutrition goals, (b) making agricultural programs and food system interventions more nutrition‐sensitive and therefore more effective in improving nutrition and health, and (c) developing capacity and leadership to use and demand appropriate evidence to improve decision‐making to this end.
